# Creating an Interest in Research and Development as a Means of Reducing the Gap between Theory and Practice in Primary Care: An Interventional Study Based on Strategic Communication

**DOI:** 10.3390/ijerph110908689

**Published:** 2014-08-26

**Authors:** Helena Morténius

**Affiliations:** 1 Department of Research and Development, Halland Hospital Halmstad, Region Halland, SE-301 80 Halmstad, Sweden; E-Mail: helena.mortenius@regionhalland.se; Tel.: +46-760-26-13-31; 2 Department of Primary Health Care, the Sahlgrenska Academy, University of Gothenburg, SE-405 30 Gothenburg, Sweden

**Keywords:** attitude, behaviour change, communication, dissemination, implementation, intervention, primary care staff, R&D interest, research capacity building (RCB), staff cohort

## Abstract

Today, healthcare professionals are faced with the challenge of implementing research results in an optimal way. It is therefore important to create a climate that is conducive to research and development (R&D). For this reason, new strategies are required to enhance healthcare professionals’ interest in innovative thinking and R&D. Strategic communication with roots in sociology, psychology and political science was employed as a means of achieving long-term behavioural change. The aim of this study was to describe, follow up and evaluate a primary care intervention based on strategic communication intended to increase healthcare professionals’ interest in R&D over time. An interventional cohort study comprising all staff members (N = 1276) in a Swedish primary care area was initiated in 1997 and continued for 12 years. The intention to engage in R&D was measured on two occasions; at 7 and 12 years. Both descriptive statistics and bivariate analyses were employed. The results demonstrated that the positive attitude to R&D increased over time, representing a first step towards new thinking and willingness to change work practices for the benefit of the patient. Strategic communication has not been previously employed as a scientific tool to create a long-term interest in R&D within primary care.

## 1. Introduction

The intention behind evidence-based care rests on well-established reasoning, but moving from the idea to behaviour is often difficult. The well-known gap between theory and practice is a major obstacle [1,2,3,4]. Considering that only a small part of new research findings and methods is actually implemented in healthcare, a greater interest in such implementation is required [5]. Studies from the USA and The Netherlands have revealed that at least every third patient fails to receive the evidence-based care that she/he needs [6,7,8]. Knowledge of the latest guidelines and new research findings in one’s own professional area is fundamental, but the implementation and use of such knowledge is frequently deemed problematic [9], one reason being that R&D appears daunting and difficult to the average practitioner [10]. This situation is more or less prevalent in the whole healthcare system and primary care is no exception. Although primary care organisations are usually long established, their readiness to engage in research is low, thus difficulties are frequently encountered when attempting to meet this demand [11]. For this reason, it is important to explore how the combination of behavioural factors, context, organisation and individuals influences the change process among primary care staff members (PCSMs) [12,13]. Research capacity building (RCB) in primary healthcare is an initiative to promote research activity among professionals and organizations in order to bridge the gap between research, policy and practice [14]. Cooke defined the function of RCB on four levels; individual, team, organisational and supra-organisation (network and support units) [15].

A preparatory phase is necessary, during which staff members actively seek knowledge of the need for critical thinking and a scientific approach. It is essential to be able to assess and reflect on which research findings to introduce, if the research has been conducted in an appropriate way, whether or not the findings are applicable to the patient group in question and last but not least, how the findings can be implemented in practice [16]. Historically, primary care research has not been prioritised and consequently interest in the field has been limited [17,18]. This lack of research interest has been linked to two important factors; the absence of both a supportive infrastructure and a facilitating research culture [19].

In Sweden, R&D in primary care was first focused on in 1970 [20]. It was introduced in family medicine and became an academic discipline in the 1980s. Based on legislation introduced in 1996, the scientific competence within healthcare was improved by the establishment of R&D units [21]. One of the aims was to promote a research culture within the healthcare system. A way of attempting to overcome existing barriers is to formulate the communication of research findings in a popular science way, as research has demonstrated that reading and assimilating a scientific article written in an unfamiliar language can constitute a barrier [2,10,22]. Another strategy is to draw attention to professionals who have assimilated research findings and adopted new methods, which means that they can serve as models and demonstrate that making use of research is neither difficult nor burdensome. A change of culture is required for primary care to become an enthusiastic and proactive research environment for all staff members, where change is driven by management, not only in terms of structural aspects such as more time and financial resources, but also by bringing about a new approach and attitude to science among all staff members [10,23].

R&D can also provide added value to the organization due to staff gaining new knowledge, becoming more involved and stimulated, which contributes to an attractive work environment [24,25]. Increased academisation is also necessary, which demands scientific competence on the part of a larger number of staff members. A long-term investment in research and innovation is required, a process that necessitates both new thinking and innovators in the field. As a learning organization, healthcare must have, among other things, R&D knowledge in order to qualify as a collaborative partner for industry, pharmaceutical companies, colleges and universities [26].

The basic concept is therefore to make R&D attractive, which can be achieved by means of strategic communication. The present author established that this method was successful in creating research interest within a short timeframe [27]. However, whether the change of attitude remains over a longer period has not been investigated.

### Strategic Communication Process

Strategic communication has been defined as “the purposeful use of communication by an organisation to fulfil its mission” [28]. It is a new interdisciplinary research field that was recognised as a scientific area in its own right in the 2000s, with application in media and communication science, business and management, sociology, political science and psychology, built on a number of theories from these areas. Strategic communication functions best when the context is open to new ideas, either in advance of or in parallel with the consolidation process [29,30]. This process can be achieved through strategically implemented communication, the strength of which is quantifiable goals based on established channels for specific target groups [31].

Strategic communication works best when individuals are willing to adopt innovative ideas prior to or during the acceptance process [30]. Consequently, it is vital that staff members who lack research experience receive the necessary support to improve their self-efficacy when dealing with research evidence. Self-efficacy is a concept within *social learning theory* [32] and concerns an individual’s perception of her/his ability to achieve various goals, thus influencing her/his motivation, choices, thoughts and attitudes. In all probability, the attitude and policy of the leadership influence staff members’ self-efficacy.

Leadership and a culture that facilitate change by promoting scientific thinking among various professional categories, especially those who are close to patients, would probably lead to a positive outcome. In addition to supporting staff to improve their self-efficacy, leadership can also mediate messages at different levels [33]. Moreover, organisations communicate discoveries and new concepts that guide leaders or others, who in turn convey them to members of the social group. Such implementation builds on a stepwise hypothesis and is the framework of the *diffusion of innovation theory* [33]. This theory, which has developed into network thinking, explains how novel ideas and findings are communicated in various social contexts over time by individuals who are influenced by different channels. It builds on assumptions regarding the adoption of information and its sequential development in the long term. Strategic communication within the studied staff cohort was partly influenced by these two theories and implemented by three established communication channels: oral, written and digital towards attitudes and work practice for the benefit of the patient. Nevertheless, studies have found that although changes in attitude can be successful, considerable barriers are involved [34]. However, what is not known is whether the change in attitude can be sustained over a longer period when the adoption process among practitioners has been gradual (implicit attitudes) [35]. The aim of the study was to describe, follow up and evaluate a primary care intervention based on strategic communication intended to increase healthcare professionals’ interest in R&D over time.

Scientific issues:

1. Can strategic communication contribute to creating interest in R&D among PCSMs over time?
2. What is the role of the direct and indirect communication channels in this context?
3. What is the role of the background variables in the development process?

## 2. Methods

### 2.1. Design and Setting

This interventional study had a prospective design aimed at influencing the attitude to a science-promoting platform, the purpose of which was to create long-term interest in R&D. The study population comprised all PCSMs in the Halland region in south west of Sweden. There are approximately 290,000 inhabitants in the region [36], including 7000 healthcare employees, of whom 20% work in primary care [37]. The population has access to state funded healthcare comprising national, regional and local levels. The regional level, where care is provided by the County Councils, constitutes the foundation for tax funded healthcare [38]. The main area of responsibility in primary care includes overall care as well as health issues that do not require specialist treatment. In a proposed amendment to the Swedish Health and Medical Act, the Swedish Government recommended in 1996 that scientific competence within the county councils should be enhanced by the creation of Research and Development units [21], resulting in the formation of a number of such units in the health service. This meant that research would be conducted outside the university hospitals, thus playing an important role in stimulating the research culture within healthcare as well as facilitating greater readiness to change in terms of assimilating new knowledge. The R&D units are generally funded by the public sector, which was also the case with the primary care R&D unit in Halland.

### 2.2. Strategic Communication Intervention

**Figure 1:**
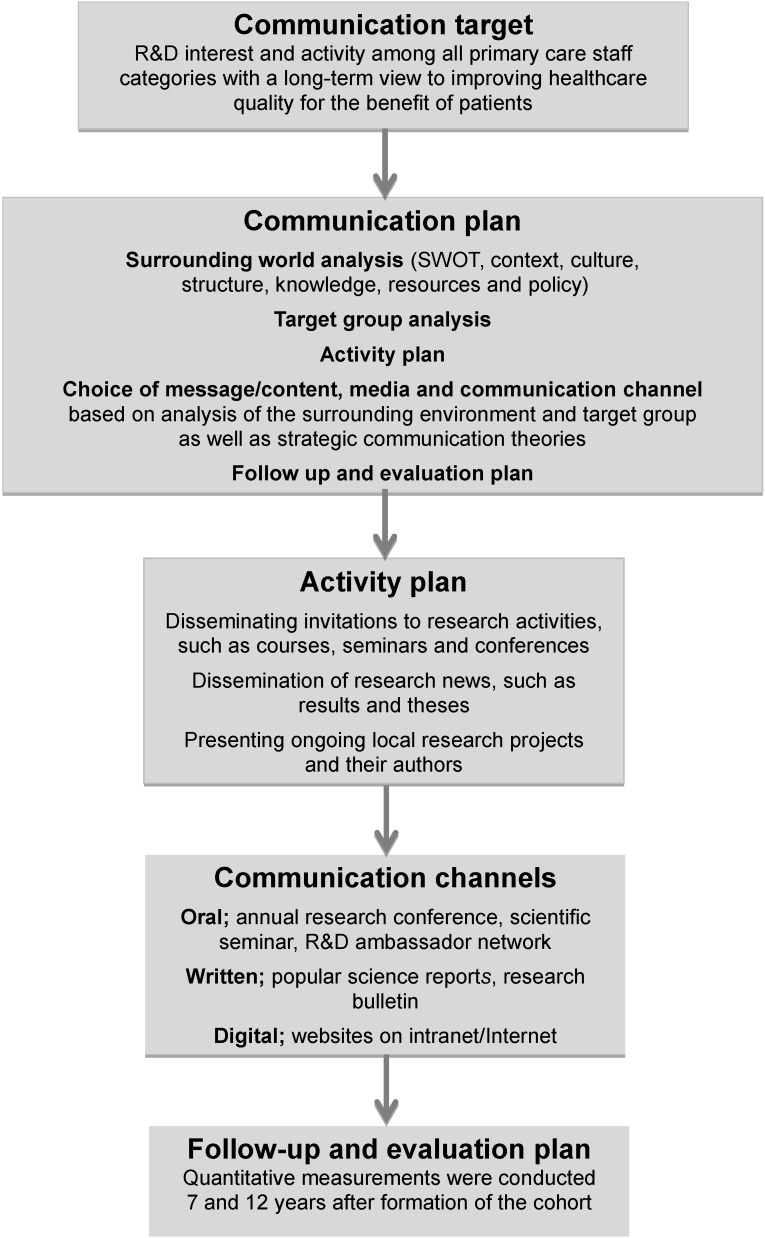
Intervention flow chart of measurement outcomes in relation to the goal of the communication strategy in the study cohort.

Note: The size of the initial study cohort (N = 1276).

The strategic communication consisted of quantifiable targets for all PCSMs in the cohort. The aim was to increase knowledge and awareness of as well as interest in R&D as a step towards promoting the creation of a new R&D culture that will generate benefit for patients over time. Initially, a communication plan was formulated on the basis of the defined target and comprised surrounding world analysis, target group analysis and an activity plan. It also contained a follow-up and a measurement flowchart to enable an overview of the long-term change in attitudes (Figure 1).

The intervention mainly involved three established communication channels: oral (research seminars and annual research days), written (research bulletins and popular science reports) and digital (intranet and Internet websites). The content of these channels was based on a communicative platform [31,32,33]. The choice of forum for dialogue was intended to increase staff self-efficacy in order to encourage PCSMs to assimilate information about R&D and incorporate it in clinical practice. The content of the seminars was adapted to the target group and had a popular science format rather than a purely scientific perspective [27,34]. The intention behind the research bulletin was to disseminate R&D evidence and news relevant to various professional categories with different educational backgrounds. The digital channel served as a complement to the oral and written channels. As R&D was a relatively new concept in the organization [39,40], focus was placed on dissemination and gaining acceptance of its importance for the personal development of each employee as well as for the organization as a whole.

### 2.3. Follow Up of the Intervention

Measurements were obtained 7 and 12 years after formation of the cohort. It was assumed that a long-term effect would reflect the impact of the intervention and not the influence of a campaign or a specific event. At the start of the study, the primary care organisation had no history of performing research, nor any systematic communication channels to the scientific community, although scientific journals were available in medical libraries. An additional factor was that all employees had access to a computer. The management in the region decided to create an R&D unit to promote the introduction of evidence-based practice (EBP) in primary care comprising scientific experts within the fields of medicine, nursing, midwifery, biostatistics, public health, behavioural science and communication science.

The result of the first measurement at seven years revealed that a majority of PCSMs had gained knowledge of R&D thanks to the various communication channels [27]. Knowledge of R&D can be considered the first step towards developing an interest in R&D and undertaking R&D activities, which corresponds with the process of changing attitudes [33,41]. More than half of the staff members who gained knowledge retained an interest in R&D [27]. An innovation process takes time as some accept new ideas more quickly than others [29]. The positive effect was increased focus on R&D, an awakening of interest among PCSMs and that R&D became a frequent topic of conversation [31,42]. However, some who were interested did not proceed further. Their responses to an open-ended question revealed that obstacles to R&D existed, which were associated with the lack of a stimulating research environment.

This led to the decision to conduct an additional study in a selected care centre by means of an ethnographic method [10]. The role of the organizational culture in creating R&D interest was illuminated by means of observations, interviews and analysis of documentation. The study revealed that theory and practice constituted two separate spheres. It was revealed that the group members firmly believed that R&D was a separate area divorced from their own. Here, management has an important role in discussing future goals with staff members. Accordingly, it was considered essential to focus the next step of the intervention on achieving integration between theory and practice using role models and opinion leaders.

The development of strategic communication continued with the establishment of a network to complement the intervention [43,44], which took place two years before the final evaluation. PCSMs who exhibited the greatest interest (early adopters) and had a basic knowledge of scientific theory and methods from their university education formed the core of the R&D ambassador network. These ambassadors represented all professional categories within primary care. The strategy was to achieve a long-term dissemination effect, first within the network itself and then among the rest of the staff. The aim of the network was twofold: (1) to enable the ambassadors to utilize their scientific knowledge to market, communicate and transfer EBP within their own department; and (2) at the same time determine the appropriate form of EBP for their own department due to being familiar with its needs. In addition to acting as research role models, the ambassadors enjoyed the benefit of becoming part of a social network and receiving research information through newsletters, meetings and further training together with like-minded colleagues [45]. The goal of the network (short-term effect) was achieved by ensuring that it served as a platform for future interventions by promoting innovative thinking.

### 2.4. Participants and Data Collection

The intervention included all PCSMs (N = 1276). Due to the long study period and the high primary care staff turnover, the participants were deemed a suitable population for the analysis. Furthermore, the proportion of SES groups that participated was equal to that of the original population at the time of the first measurement [27]. A total of 846 employees (70%) took part in the measurement (short term) and at the 12-year follow up (long term) the participation rate was 60%. Dropouts were non-responders, those who declined participation for various reasons, among other things parental and sick leave or due to incomplete questionnaires as well as those who no longer worked in primary care (n = 362). Furthermore, those individuals who worked at the R&D units as well as the personnel who recruited the pilot group were not included (n = 70). The responses in this study were obtained from a questionnaire that was sent on two occasions followed by two reminders.

All participants were invited to complete the questionnaire voluntarily and their confidentiality was guaranteed. The study conformed to the principles outlined in the Declaration of Helsinki [46] and was approved by the Ethics Committee at Lund University, Sweden.

### 2.5. Instrument and Statistical Methods

The questionnaire was designed to determine the influence of R&D related communication on changes in PCSMs’ attitudes to R&D over time. The items were formulated by a research team composed of a communications expert, a researcher in the area of healthcare communication, healthcare research experts and a biostatistician [27]. The items covered background (*age,* *sex* and *profession*) and *years of practice* in addition to *R&D interest* as an effect of strategic communication. There were 16 items on the role of strategic communication through direct and indirect channels and two items were added that pertained to whether or not *the R&D ambassadors had created a distinctive image for themselves among the staff.*

Items pertaining to professional status were translated into socio-economic status (SES) using the National Socio Economic Dictionary [47]. The SES groups were divided into four sub-groups: I: Assistant nurse. II: Dental nurse (assistant), medical secretary, administrative staff. III: Nurse, district nurse, midwife, dental hygienist, physiotherapist, occupational therapist. IV: Physician, dentist, psychologist. The SES sub*-*groups were divided according to a ranking taking account of the social position of a profession with emphasis on educational level.

The responses were processed using the SPSS 21.0 [48]. Descriptive statistics and bivariate analysis using Chi-square were employed in the calculation [49]. The level of significance was set at 0.05. To illustrate the impact of age on the likelihood of R&D interest in each SES group, logistic regression analysis was performed. A beta-coefficient and *p*-value as well as an odds ratio (OR) and a 95% confidence interval (CI) were calculated as a confirmation of the statistical significance [50].

## 3. Results

The intervention took place over a 12-year period and included two measurements (measurement I: n = 846; measurement II: n = 762). Most of the participants were female (measurement I: 89%; measurement II: 90%). Their mean age was 49.5 (SD = 8.8) on the first measurement occasion and 49.6 (10.1) on the second. The largest number of participants belonged to the SES III category (n = 376 and n = 340 respectively) which corresponded to 45.8% on the first measurement and 44.6% on the second (Table 1).

**Table 1:** Descriptive statistics pertaining to the distribution of background variables.

Study Variables	First Measurement				Second Measurement			
	Year 2004				Year 2009			
	n	%	Mean (SD)	Median (IQR)	n	%	Mean (SD)	Median (IQR)
Sex	846	**100.0**			762	**100.0**		
Men	95	11.2			73	9.6		
Women	751	88.8			688	90.4		
Age	846	**100.0**	49.5	50.0	757	**100.0**	49.6	51.0
			(8.8)	(44–56)			(10.1)	(51–58)
SES	820	**100.0**			730	100.0		
I	71	8.7			50	6.8		
Assistant nurse	71	8.7			50	6.8		
II	205	25.0			185	25.3		
Dental nurse (assistant)	104	12.7			103	14.1		
Medical secretary	51	6.2			52	7.1		
Administrative staff	50	6.1			30	4.1		
III	376	45.8			340	44.6		
Nurse	51	6.2			47	6.4		
District nurse	159	19.4			129	17.6		
Midwife	36	4.4			40	5.4		
Dental hygienist	38	4.6			32	4.6		
Physiotherapist	56	6.8			55	7.5		
Occupational Therapist	36	4.4			35	4.7		
IV	168	20.5			155	20.3		
Physician	77	9.4			65	8.9		
Dentist	63	7.7			58	7.9		
Psychologist	28	3.4			32	4.4		

Note: n = number of participants in the analysis.

At the first measurement, the interest created by means of the R&D unit’s strategic communication channels was significantly higher compared to external channels (60.2% *vs.* 39.8%; *p* < 0.0001). After a further five years the positive attitude towards R&D had improved and stabilised (69.1% *vs.* 30.9%; *p* < 0.0001). Moreover, there was a statistically significant relationship between R&D interest and knowledge of the R&D ambassadors in the field (*p* < 0.05). On the whole, the channels employed in the strategic communication played a vital role in terms of consolidating R&D interest.

**Figure 2:**
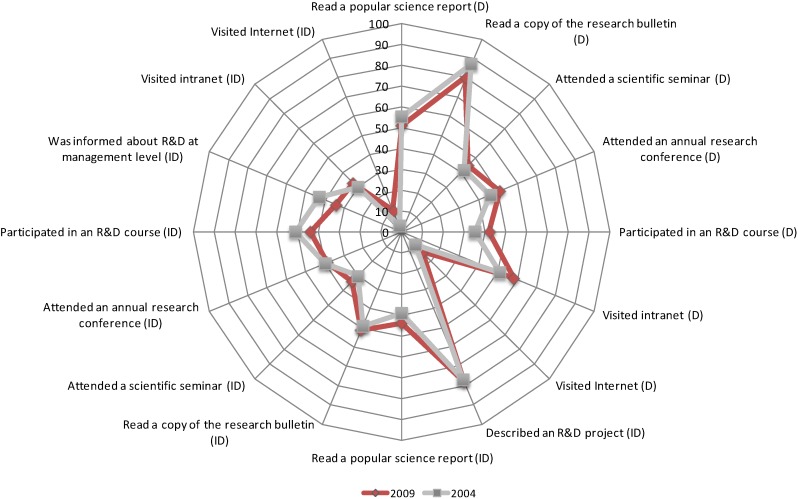
Impact of the direct (D) and indirect communication (ID) communication channels on the creation of interest in R&D among primary care staff measured on two occasions.

Note: 100 represents maximal impact.

Staff members’ own initiative to read the research bulletin was found to have the greatest impact on R&D interest on both occasions (*p* < 0.001). In addition, indirect communication, *i.e.*, a colleague narrating about various R&D projects (*p* < 0.001), a scientific seminar (*p* < 0.001) and an annual research conference (*p* < 0.01), contributed substantially to individual interest in R&D (Table 2). The influence of the digital channels was negligible despite the fact that the results were significant on both occasions (*p* < 0.001) (Table 2). When occasions I and II were analysed, the channels’ contributions in terms of creating an interest in R&D were more or less the same (Figure 2).

**Table 2:** Gained interest in R&D as a result of direct and indirect communication.

The Different Communication Ways	Gained R&D Interest 2004					Gained R&D Interest 2009			
	n	%	chi-2	*p*		n	%	chi-2	*p*
***Through one’s own initiative (direct)***									
Read a popular science report	198	54.5	1.64	0.201		184	51.5	0.09	0.768
Read a copy of the research bulletin	270	87.0	148.15	<0.001		252	80.2	91.68	<0.001
Attended a scientific seminar	190	42.1	4.74	0.030		182	44.5	2.20	0.138
Attended an annual research conference	211	46.4	1.07	0.302		205	51.2	0.12	0.727
Participated in an R&D course	195	34.9	17.85	<0.001		200	42.0	5.12	0.024
Visited intranet	211	51.2	0.12	0.731		206	57.8	4.97	0.026
Visited Internet	153	8.5	105.42	<0.001		156	13.5	83.31	<0.001
***Heard about somebody who had (indirect)***									
Described an R&D project	246	76.8	70.83	<0.001		228	78.1	71.86	<0.001
Read a popular science report	163	38.7	8.40	0.004		164	43.9	2.44	0.118
Read a copy of the research bulletin	172	48.8	0.09	0.760		182	50.5	0.02	0.882
Attended a scientific seminar	154	29.9	24.96	<0.001		147	34.0	15.03	<0.001
Attended an annual research conference	166	39.8	6.96	0.008		159	39.0	7.70	0.006
Participated in an R&D course	174	50.6	0.02	0.879		161	43.5	2.74	0.098
Been informed about R&D at management level	162	43.2	2.99	0.084		152	34.2	15.16	<0.001
Visited intranet	163	30.1	25.92	<0.001		154	32.5	18.94	<0.001
Visited Internet	141	2.8	125.45	<0.001		142	11.3	85.21	<0.001

Notes: The measurements took place on two occasions; 2004 and 2009. The chi-square test was employed.

Each SES group was analysed separately in order to investigate whether or not the variable age had an impact on the probability of change in increased interest in R&D interest. The results of the logistic regression indicated a significant effect of the variable age on R&D interest in SES groups III (OR 0.979; 95% CI 0.958–0.988) and IV (OR 0.950; 95% CI 0.924–0.976) meaning that those with a higher SES had a rapidly decreasing probability of interest, while those with a lower SES had a low probability of having an interest also at high ages (Table 3).

**Table 3:** The influence of the variable age on R&D interest in four separate SES groups.

Dependent Variable: R&D Interest		Independent Variable: Age				
1= Interest						
0= No Interest						
	**N**	**Beta-coefficient**	***p*-value**	**OR**	**95% CI**
**SES I**	121	−0.09	0.257	0.917	0.789–1.065
**SES II**	382	−0.01	0.792	0.993	0.942–1.047
**SES III**	716	−0.02	0.047	0.979	0.958–0.988
**SES IV**	323	−0.05	<0.0001	0.950	0.924–0.976

Notes: R&D interest: Difference between the 2004 and 2009 measurements. Logistic regression was used. An Odds Ratio (OR) and 95% Confidence Interval (CI) were included in the calculation.

**Figure 3:**
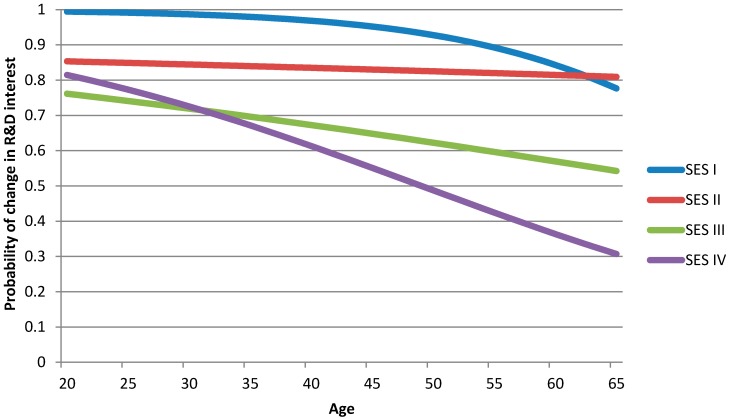
The probability of change in R&D interest as a function of age.

Note: A separate analysis was performed for each SES group by the use of logistic regression.

R&D interest was highest in younger age groups but decreased in line with increasing age in all SES groups with the exception of SES II. This result was statistically significant for SES III and SES IV (*p* < 0.05). The interest decreased more rapidly in SES IV than SES III, where the breakpoint occurred at age 32 (Figure 3).

## 4. Discussion

### 4.1. Long-Term Interest in R&D

In the mid-1990s, R&D units were not common or well-established within healthcare organisations. Thus our strategy was to make the individual PCSM familiar with R&D. Our choice of media strategy was motivated by the intention of reaching all PCSMs in order for R&D to become a daily topic of conversation in primary care centres. Many strategies to introduce new knowledge are directed towards individual professional categories, such as nurses or doctors or a combination of both [51,52,53,54,55]. Although the importance of multidisciplinary and interprofessional efforts has been highlighted [56], the role of the organisation as a whole has not been described to the same extent [57]. By targeting all professional categories in the organisation and creating interest, every PCSM can feel involved and engage in the discussions taking place on a daily basis. The involvement of different staff categories in interventions that concern the whole care chain is recommended from a practical as well as a promotional patient benefit perspective [58,59,60]. In the implementation phase, it is essential that both intention and interest are sustained.

It is a well-known fact that creating an interest and changing attitudes take time [29]. Thus, the communication strategy implemented twelve years earlier focused on a long-term change in attitudes. It was essential to devote time and effort to creating an interest, which would then serve as a platform for more advanced aspects of the research process [61]. An advantage of long-term intention is that it creates stability of attitudes, which is considered to promote the formation of an organisational culture [62]. However, it should be borne in mind that during the intervention, unknown confounders are likely to play a role and influence the net effect.

### 4.2. The Role of the Communication Channels

The choice of communication channels proved successful, as the resulting interest was almost identical when measurements I and II were compared. Among those who became involved in the activities on their own initiative, the effect was anticipated, as they probably already had an interest in R&D. However, the greatest benefit could be seen among those who became interested in an indirect way and visited the channels due to reports from others [31]. The greatest influence in terms of interest was seen among those who had read the research bulletin and those who had heard a colleague describe an R&D project.

Among other things, increased interest in R&D was consolidated by means of PCSMs who had been involved in R&D activity. These can be seen as role models for different professional categories, not least in the written channel, which was read by nine out of ten PCSMs [27]. The research bulletin was intended to create awareness of R&D in a popular science form, which several studies have highlighted as necessary for disseminating the message [22,63]. The strategic approach of employing the written channel to create an interest in other channels, thus contributing to a synergy effect, is a well-known phenomenon in the context of communication [64]. A strategy to attract readers was using the research bulletin as a medium for invitations to new courses, lectures and research days in addition to reports about such days that included photos and news. The strategy of placing a number of bulletins in the staff room to increase the likelihood that they would become a topic of conversation, rather than sending them to the home address of each PCSM, probably contributed to the change in attitude. The fact that the digital channels were not successful in this respect is most likely due to the use of the digital medium, which was limited to the handling of patient records and similar.

### 4.3. Colleagues and Manager Create Interest

The indirect communication channels continued to have a long-term effect. This was achieved by colleagues who told about their R&D projects and were given a distinctive image as role models, which result is supported by previous studies [65]. The researchers or course participants who obtained R&D information in different ways disseminated it to their target groups in accordance with a two-step hypothesis. This is to a great extent in line with Shaw (2004), who suggests that enthusiastic and committed individuals can drive research due to their ability to influence others, as well as co-ordinate activities and teams across departmental and organisational boundaries [66]. Mentoring has been identified as a key element of training and development because of its importance in supporting young researchers to develop research skills before they become overwhelmed by other demands [67,68]. In this context, the role of management is often described as a major factor in the creation of a good research climate in an organisation [23]. This is supported by our study, where a third of the PCSMs had become interested after their manager had informed them about R&D.

### 4.4. The influence of Networks

Over the course of almost two years, the R&D ambassadors created a distinctive profile for themselves within the context. They formed a network with a dual role; encouraging individual PCSMs to engage in R&D activities and serving as role models. Previous studies have described and explored the significance of networks for new innovations [44,69,70]. It can therefore be assumed that the R&D ambassador network had a positive influence by forming a bridge between the theoretical-scientific world and clinical practice. The ethnographic study [10] found that in many cases the PCSMs were fairly isolated and did not use R&D to any great extent. The ambassadors are able to identify the need for R&D within their own unit as well as how best to introduce innovative thinking. Willingness to change is enhanced when a familiar person promotes and pushes an individual or a group towards higher self-efficacy [32]. In the long term, it can contribute to greater willingness in organisational cultures that are slow to accept change.

The importance of networks for integrating academic and service initiatives has been discussed [71,72,73,74]. Different types of organization are associated with various network profiles, which is a consequence of the collaboration partners available. A common feature is, however, the necessity of carefully preparing the network over a longer period and the ambassador network is no exception. The ambassador network in our study had been prepared by means of advanced courses, lectures, their own web-based magazine and website, thus providing knowledge that they could communicate to their colleagues over time. It is also important that the networks can build capacity from the bottom up [71], where the members themselves can motivate and support each other through network meetings and at education sessions. The role of the network members enables them to adopt a top-down approach by legitimizing and creating research resources for their colleagues, as well as working from the bottom up where they recognize the need for research within the organisation and raise the topic in their own group. All PCSMs must be involved in order to promote the integration of scientific thinking in the health services [75].

### 4.5. Interest in R&D Is Greatest among Younger Staff Members

Socialization into the organization is dictated by the organizational culture, despite strong social and socio-demographic affiliation, and is eventually taken for granted, which means that internalization has occurred [76]. Professional roles and socialization are two factors that must be considered in a hierarchical organization such as the health services [77]. Studies comprising nurses as well as physiotherapists have established that the socialization process only took two to five years after completion of the education at which time their use of research evidence dropped drastically [78,79,80]. This means that extra resources are required to utilize younger PCSMs interested in research, thus promoting research within primary care. Research carried out with occupational therapists and physiotherapists revealed that individuals with higher degrees are more likely to have the ability to generate clinical research questions, search databases, understand research terminology and be more confident in undertaking these tasks [79,81,82], which benefits the organization.

### 4.6. General Discussion

The aim was to create a long-term interest in R&D by means of a strategic communication intervention among all PCSMs. The shift in paradigm that has taken place within primary care can form a basis for discussion about future interventions that also include other dimensions of RCB [15].

The present study describes how RCB was promoted at the individual level, where recently graduated staff members were encouraged by their managers to maintain their innovative thinking, thereby influencing their own organization. However, the organization needs an overarching R&D policy to support managers in implementing an evidence-based mode of working. Here, the role of R&D units and similar departments is central in providing an infrastructure for the creation of a sustainable research culture where leadership is exercised through mentoring, role models and where researchers promote research and actively disseminate research results [68,83]. This accords with the strategic work presented in the present study; the importance of the research culture supporting scientific thinking, learning and further training in addition to the communication intervention promoting new thinking and interest in both internal and external R&D findings and news.

The barriers are well known, namely the individuals’ lack of self-efficacy belief in terms of their ability to work on the basis of research evidence as well as the structural and especially the cultural circumstances that more or less determine the possibility to work in an evidence-based manner. A process over a longer period characterized by implicit learning [35] can lead to changed attitudes among those who were not early adopters. Implicit attitudes are much slower to change, which is consistent with a slow-learning [35]. Working strategically with a communication intervention that involves constant follow up over a longer period aimed at providing a foundation for building future R&D commitment is challenging. Despite their obvious limitations in capturing the complexity of organizational development, phase models are useful for highlighting the underestimated role of time in understanding change [84].

In a hierarchical organization, the frameworks and organizational policy are in place, making it difficult to influence the culture [65,85,86]. Groups consisting of different professional categories are considered an advantage when new attitudes are introduced [58,59]. Although the formation of teams is seen as promising in terms of learning, difficulties and obstacles exist when it comes to values, perspectives, understanding and role conflicts [57], often in combination with organizational obstacles constituted by hierarchical levels within the healthcare system [87]. However, the role of the organizational culture as a whole in the visualisation of goals is not discussed to any great extent in the literature.

When management is prepared to change the existing culture, a more reflective insight into the staff members’ attitudes is required [88], especially the ‛taken for granted’ attitudes in the organizational culture [89]. A dialogue is also necessary between management and staff members about the underlying attitudes, norms and values that govern planning, decision-making and actions [10], which require greater focus when introducing new thinking and methods, such as scientific thinking and R&D intention. Where the culture and strategies are not aligned, organizations may lose valuable employees who have the critical skills and experience to move easily to another organization [90].

A major organizational change was introduced in Sweden in 2007 allowing people the right to choose a primary care centre irrespective of their place of residence [91]. This led to competition between care centres and an acceleration of care production. When care centres are exposed to competition and each crown spent on patients is weighed against other activities, R&D can probably be a burden as the concrete benefit is not immediately obvious. A possible long term consequence is that a primary care centre that lacks research and development jeopardizes its own credibility, leading to inferior quality throughout the health services [58]. A culture that enables staff members to engage in research enhances the work environment and promotes personal development. This in turn will make the organization competitive, thus providing an edge in its marketing strategy directed towards the well informed patients of today. The present study comprised all primary care professional categories, which constitutes a strength when investigating overall changes in attitudes. The study was also strengthened by the choice of design where all participants took part in a second measurement. Moreover, the long-term intervention was a strength, as it made it possible to study whether or not changes in attitude were maintained over time.

More and more research is being carried out and an increasing number of studies are published, a development that can be assumed will continue and perhaps even accelerate, but what stance will practitioners take in relation to this? Can interest in R&D combined with critical thinking and a scientific approach on the part of practitioners and researchers be seen as the first step towards taking part in the discussion, making their voices heard within their own organization, as well as at national and international level? The goal of primary care should not be to make everybody a researcher but to provide all staff members with the opportunity to be good research consumers.

### 4.7. Limitations

One limitation is the fact that the accuracy of the study would have been improved had it been possible to follow every individual over time (longitudinal) to decrease the existence of selection bias and increase the reliability of the study. Another limitation is that, the study would have benefited from the inclusion of a control group. However, this was not possible due to differences between Swedish primary care areas. Finally, the third limitation was that the adequate baseline information would have decreased the limitation of the study but an analysis of the general state of the research culture in the context under study conducted by the Region Halland (County Council) shortly before the intervention revealed that it lacked an R&D tradition [40].

## 5. Conclusions

Strategic communication has not been previously employed as a scientific tool to create a long-term interest in R&D within primary care. The results demonstrated that strategic communication led to an increased interest in R&D that was sustained over time. This finding represents a first step towards research intention and capacity building for the benefit of the patient. The direct and the indirect communication channels as well as the oral and the written channel played a significant role in the improvement process, which can assumed to have contributed to increasing the synergy effect on the staff members’ positive attitude to R&D. The positive attitude appeared to be most evident among SES groups III and IV as well as among younger staff, which is beneficial, as these groups play an important role in reducing the gap between theory and practice.
